# Author Correction: Pathway level subtyping identifies a slow-cycling biological phenotype associated with poor clinical outcomes in colorectal cancer

**DOI:** 10.1038/s41588-024-01809-4

**Published:** 2024-06-03

**Authors:** Sudhir B. Malla, Ryan M. Byrne, Maxime W. Lafarge, Shania M. Corry, Natalie C. Fisher, Petros K. Tsantoulis, Megan L. Mills, Rachel A. Ridgway, Tamsin R. M. Lannagan, Arafath K. Najumudeen, Kathryn L. Gilroy, Raheleh Amirkhah, Sarah L. Maguire, Eoghan J. Mulholland, Hayley L. Belnoue-Davis, Elena Grassi, Marco Viviani, Emily Rogan, Keara L. Redmond, Svetlana Sakhnevych, Aoife J. McCooey, Courtney Bull, Emily Hoey, Nicoleta Sinevici, Holly Hall, Baharak Ahmaderaghi, Enric Domingo, Andrew Blake, Susan D. Richman, Claudio Isella, Crispin Miller, Andrea Bertotti, Livio Trusolino, Maurice B. Loughrey, Emma M. Kerr, Sabine Tejpar, S:CORT consortium, Timothy S. Maughan, Mark Lawler, Andrew D. Campbell, Simon J. Leedham, Viktor H. Koelzer, Owen J. Sansom, Philip D. Dunne

**Affiliations:** 1 The Patrick G Johnston Centre for Cancer Research, Queen’s University Belfast, Belfast, UK; 2 Department of Pathology and Molecular Pathology, University Hospital Zurich, University of Zurich, Zurich, Switzerland; 3 Faculty of Medicine, Université de Genève, Geneva, Switzerland; 4 Cancer Research UK Scotland Institute, Glasgow, UK; 5 Centre for Human Genetics, University of Oxford, Oxford, UK; 6 Candiolo Cancer Institute, FPO IRCCS, Candiolo, Torino Italy; 7 Department of Oncology, University of Torino, Candiolo, Torino Italy; 8 School of Electronics, Electrical Engineering and Computer Science, Queen’s University Belfast, Belfast, UK; 9 Department of Oncology, University of Oxford, Oxford, Oxfordshire UK; 10 Leeds Institute of Medical Research, University of Leeds, Leeds, UK; 11 School of Cancer Sciences, University of Glasgow, Glasgow, UK; 12 Department of Cellular Pathology, Royal Victoria Hospital, Belfast Health and Social Care Trust, Belfast, UK; 13 Department of Oncology, Katholieke Universiteit Leuven, Leuven, Belgium

**Keywords:** Colon cancer, Tumour biomarkers

Correction to: *Nature Genetics* 10.1038/s41588-024-01654-5, published online 13 February 2024

In the version of the article initially published, the S:CORT consortium was missing from the author list and has now been added to the HTML and PDF versions of the article.

