# Supplementary material for: Pathway level subtyping identifies a slow-cycling biological phenotype associated with poor clinical outcomes in colorectal cancer
Source: Nat Genet. 2024 Feb 13;56(3):458–72. doi: 10.1038/s41588-024-01654-5 (PMC10937375; doi:10.1038/s41588-024-01654-5)
Supplement: Supplementary file 1 — List of consortia members [file 41588_2024_1654_MOESM1_ESM.pdf]

nature genetics

Supplementary information https://doi.org/10.1038/s41588-024-01654-5

Pathway level subtyping identifies a slow-cycling biological phenotype associated with poor clinical outcomes in colorectal cancer

In the format provided by the authors and unedited

The S:CORT consortium membership includes: Andrew Blake, Francesca Buffa, Enric Domingo, Geoff Higgins, Chris Holmes, Viktor Koelzer, Simon Leedham, Timothy Maughan, Gillies McKenna, James Robineau, Ian Tomlinson and Michael Youdell from the University of Oxford; Philip Quirke, Susan Richman, David Sebag-Montefiore, Matthew Seymour and Nicholas West from the University of Leeds; Philip Dunne, Richard Kennedy, Mark Lawler, Keara Redmond and Manuel Salto-Tellez from Queens University Belfast; Peter Campbell, Aikaterina Chatzpili, Claire Hardy and Ultan McDermott of the Wellcome Trust Sanger Institute; Simon Bach, Andrew Beggs, Jean-Baptiste Cazier, Gary Middleton, Dion Morton, Celina Whalley from the University of Birmingham; Louise Brown and Richard Kaplan from University College London; Graeme Murray from the University of Aberdeen; Richard Wilson, University of Glasgow; Richard Adams University of Cardiff; Richard Sullivan, Kings College London, Les Samuel, Brampian NHS Health Board; Paul Harkin and Steven Walker of Almacgroup; Jim Hill of Christie Hospital Manchester; Denis Horgan, European Association of Precision Medicine; Chieh-Hsi Wu, Southampton University.
