# Supplementary material for: Pathway level subtyping identifies a slow-cycling biological phenotype associated with poor clinical outcomes in colorectal cancer
Source: Nat Genet. 2024 Feb 13;56(3):458–72. doi: 10.1038/s41588-024-01654-5 (PMC10937375; doi:10.1038/s41588-024-01654-5)
Supplement: Supplementary file 2 — Reporting Summary [file 41588_2024_1654_MOESM2_ESM.pdf]

nature portfolio

Corresponding author(s): Philip Dunne

Last updated by author(s): Oct 27, 2023

Reporting Summary

Nature Portfolio wishes to improve the reproducibility of the work that we publish. This form provides structure for consistency and transparency in reporting. For further information on Nature Portfolio policies, see our Editorial Policies and the Editorial Policy Checklist.

Statistics

For all statistical analyses, confirm that the following items are present in the figure legend, table legend, main text, or Methods section.

n/a	Confirmed	
☐	☒	The exact sample size (*n*) for each experimental group/condition, given as a discrete number and unit of measurement
☐	☒	A statement on whether measurements were taken from distinct samples or whether the same sample was measured repeatedly
☐	☒	The statistical test(s) used AND whether they are one- or two-sided<br>*Only common tests should be described solely by name; describe more complex techniques in the Methods section.*
☐	☒	A description of all covariates tested
☐	☒	A description of any assumptions or corrections, such as tests of normality and adjustment for multiple comparisons
☐	☒	A full description of the statistical parameters including central tendency (e.g. means) or other basic estimates (e.g. regression coefficient) AND variation (e.g. standard deviation) or associated estimates of uncertainty (e.g. confidence intervals)
☐	☒	For null hypothesis testing, the test statistic (e.g. *F*, *t*, *r*) with confidence intervals, effect sizes, degrees of freedom and *P* value noted<br>*Give P values as exact values whenever suitable.*
☒	☐	For Bayesian analysis, information on the choice of priors and Markov chain Monte Carlo settings
☒	☐	For hierarchical and complex designs, identification of the appropriate level for tests and full reporting of outcomes
☐	☒	Estimates of effect sizes (e.g. Cohen's *d*, Pearson's *r*), indicating how they were calculated

Our web collection on statistics for biologists contains articles on many of the points above.

Software and code

Policy information about availability of computer code

Data collection

A combination of both public and proprietary gene expression datasets (both microarray and RNA-Seq) were used for the study. The publicly available gene expression datasets were downloaded and are available at Gene Expression Omnibus (GEO) with the accession number reported in the study as: GSE156915 (FOCUS), GSE39582, and GSE31279 (laser-captured microdissected dataset). RNA-Seq data includes TCGA Colon and Rectal Adenocarcinoma (COREAD) downloaded as HT-Seq counts from Genomic Data Commons (GDC) via TCGAbiolinks R package (v2.16.1); GSE143915 via GEO (mouse intestinal crypt dataset). The genetically engineered mouse model (GEMM) primary tumour dataset can be accessed via the accession code GSE218776 (RNA-Seq). In the case of analyses with the data from PETACC-3 trial (NCT00026273), it was analysed by Dr Petros Tsantoulis and overseen by Prof Sabine Tejpar.

Other proprietary expression dataset (S:CORT consortium) includes: SPINAL (CRC cohort; microarray), and polyp dataset (RNA-Seq) will be disclosed prior to the publication. These datasets will be released in collaboration with a Cancer Research UK data access committee, who are committed to supporting the FAIR principles and to ensure use of these cohorts for academic researchers.

Processed count expression matrices from previously published single cell RNA sequencing data of CRC patient samples (Joanito et al., 2022, Nature Genetics) were requested and downloaded through Synapse (syn26844071). Single cell dataset derived from murine organoid models from Qin and Cardoso Rodriguez et al., 2023, BioRxiv) was downloaded from Zenodo (10.5281/zenodo.7586958). The datasets included in the study will be further made available prior to the publication.

Data analysis

We used R (v4.1.2) and RStudio (v2022.09.2.382), QuPath (v0.2.3) and SideFX Houdini 19.5 has been utilised in the study. All the software versions (including R-related packages) have been provided in the "Method" section.

Statistical analysis conducted in this study has been performed in R using stats (v4.2.1) or ggpubr (v0.4.0) R package for plots, including two-

sided Wilcoxon rank-sum test, Kruskal-Wallis rank-sum test, Fisher's exact test, and Pearson's correlation coefficient test. For copy number by arm analysis, Pearson' Chi-squared test post-hoc analysis was performed using chisq.posthoc.test R package (v0.1.2) and adjusted P-value with Benjamini-Hochberg using p.adjust function in stats R package. Other R packages that have been utilised for data analysis and data visualisation include, ggtern (v3.3.5), ComplexHeatmap (v2.10.0), circlize (v0.4.15), umap (v0.2.8.0), ggplot2 (v3.3.6), patchwork (v1.1.1), riverplot (v0.10), ggforce (v0.3.3), RColorBrewer (v1.1-2).

The PDSclassifier R package (v0.1.0) is available on the Molecular Pathology Lab GitHub (https://github.com/MolecularPathologyLab/PDSclassifier). All new data and code will be made available prior to publication, and all the scripts related to the article will be made available on our website (www.dunne-lab.com).

For manuscripts utilizing custom algorithms or software that are central to the research but not yet described in published literature, software must be made available to editors and reviewers. We strongly encourage code deposition in a community repository (e.g. GitHub). See the Nature Portfolio guidelines for submitting code & software for further information.

Data

Policy information about availability of data

All manuscripts must include a data availability statement. This statement should provide the following information, where applicable:

- Accession codes, unique identifiers, or web links for publicly available datasets
- A description of any restrictions on data availability
- For clinical datasets or third party data, please ensure that the statement adheres to our policy

In addition to the statement below, we have also included within the Methods section detailing access points for all cohorts used. This is alongside links/references to all methods/scripts used for computational analyses throughout.

Data and Code Availability.
The PDSclassifier R package (v0.1.0) is available on the Molecular Pathology Lab GitHub (https://github.com/MolecularPathologyLab/PDSclassifier). All new data and code will be made available prior to publication, and all the scripts related to the article will be made available on our website (www.dunne-lab.com).

Also, the datasets included in the study will be also further made available prior to the publication.

The FOCUS (GSE156915) and SPINAL datasets were generated within the S:CORT programme, where microarray gene expression profiles, mutation, clinical, immunohistochemistry (IHC), tissue blocks and tumour microarrays (TMAs) were available. FOCUS: MRC-funded randomised trial cohort consisting of 360 formalin-fixed paraffin-embedded (FFPE) primary tumour samples for metastatic CRC. SPINAL: 258 FFPE samples from CRC patients, mixed stages.

The data in this publication generated by the S:CORT Consortium is available for use by not-for-profit organisations for academic, teaching and educational purposes. Gene expression profiles for the S:CORT-led SPINAL has been made available at GEO with GSEXXX. The data is available for commercial use, on commercial terms, via Cancer Research Horizons https://www.cancerresearchhorizons.com/.

Other publicly available datasets were accessed from Gene Expression Omnibus (GEO) with accession number: GSE39582, GSE31279, GSE143915, GSE218776, and from ArrayExpress E-MTAB-6363. The validation of clinical association was carried out PETACC-3 cohort. The Cancer Genome Atlas (TCGA) dataset for Colon and Rectal Adenocarcinoma (COREAD), was accessed and extracted from the Genomic Data Commons (GDC) via TCGAbiolinks.

Two epithelial single-cell RNA sequencing datasets were also utilised for the study – a CRC tissue derived scRNA-seq merged datasets from five different cohorts, and a scRNA-seq dataset derived from murine organoids mono-/co-cultured with fibroblast and/or macrophages. For the scRNA-seq human CRC dataset, the processed count expression matrix for n=49,155 epithelial cells and the corresponding epithelial metadata were downloaded through the Synapse under the accession code syn26844071. The murine organoid scRNA-seq dataset consists of n=29,452 epithelial cells from wild-type mouse colonic organoids and at least 5 different genotypic CRC organoids, including shApc (A), KrasG12D/+ (K), shApc and KrasG12D/+ (AK), KrasG12D/+ and Trp53R172H/- (KP) and shApc, KrasG12D/+ and Trp53R172H/- (AKP), and all the corresponding metadata were also downloaded from Qin and Cardoso Rodriguez et al.

Research involving human participants, their data, or biological material

Policy information about studies with human participants or human data. See also policy information about sex, gender (identity/presentation), and sexual orientation and race, ethnicity and racism.

Reporting on sex and gender	*NA*
Reporting on race, ethnicity, or other socially relevant groupings	*NA*
Population characteristics	*NA*
Recruitment	*NA*
Ethics oversight	*NA*

Note that full information on the approval of the study protocol must also be provided in the manuscript.

Field-specific reporting

Please select the one below that is the best fit for your research. If you are not sure, read the appropriate sections before making your selection.

☒ Life sciences ☐ Behavioural & social sciences ☐ Ecological, evolutionary & environmental sciences

For a reference copy of the document with all sections, see nature.com/documents/nr-reporting-summary-flat.pdf

Life sciences study design

All studies must disclose on these points even when the disclosure is negative.

Sample size	All samples sizes are indicated throughout the manuscript. No sample size calculation was performed.
Data exclusions	As mentioned in the "Method" section, for class discovery, only KRAS-mutant primary CRC tumours were selected (n=165) with KRAS wildtypes, BRAF-mutants, HRAS-mutants and NRAS-mutants excluded. The rationale being the focus of the study initially was KRAS-mutant stratification, thus exclusion of respective samples.
Replication	Following class discovery, a number of cohorts were used to validate/replicate these findings. Details of the cohorts used are in the data/code sections of this report and detailed in methods of the manuscript. The validation was done in at least three different cohorts: FOCUS, GSE39582, and SPINAL with biological findings remaining consistent across all three cohorts.
Randomization	NA
Blinding	NA

Behavioural & social sciences study design

All studies must disclose on these points even when the disclosure is negative.

Study description	NA
Research sample	NA
Sampling strategy	NA
Data collection	NA
Timing	NA
Data exclusions	NA
Non-participation	NA
Randomization	NA

Ecological, evolutionary & environmental sciences study design

All studies must disclose on these points even when the disclosure is negative.

Study description	NA
Research sample	NA
Sampling strategy	NA
Data collection	NA
Timing and spatial scale	NA
Data exclusions	NA
Reproducibility	NA
Randomization	NA

Blinding	NA

Did the study involve field work? ☐ Yes ☐ No

Field work, collection and transport

Field conditions	NA
Location	NA
Access & import/export	NA
Disturbance	NA

Reporting for specific materials, systems and methods

We require information from authors about some types of materials, experimental systems and methods used in many studies. Here, indicate whether each material, system or method listed is relevant to your study. If you are not sure if a list item applies to your research, read the appropriate section before selecting a response.

Materials & experimental systems

n/a	Involved in the study
☐	☒ Antibodies
☒	☐ Eukaryotic cell lines
☒	☐ Palaeontology and archaeology
☐	☒ Animals and other organisms
☐	☒ Clinical data
☒	☐ Dual use research of concern
☒	☐ Plants

Methods

n/a	Involved in the study
☒	☐ ChIP-seq
☒	☐ Flow cytometry
☒	☐ MRI-based neuroimaging

Antibodies

Antibodies used	*Ki67, Chromogranin A, Synaptophysin, Hs-ANXA1 (465411), and Hs-LGR5-C2 (311021-C2).*<br><br>*BrdU (BD Biosciences 347580, TRS High, 1:250), Ki67 (Cell Signalling 12202, ER2 20min, 1:1000), Chromogranin-A (AbCam ab108388, TRS High, 1:600) and Synaptophysin (Cell Signalling 36406, TRS High, 1:150)*
Validation	*Previously developed and validated by Ester Gil Vasquez et al., https://pubmed.ncbi.nlm.nih.gov/35931031/*

Eukaryotic cell lines

Policy information about cell lines and Sex and Gender in Research

Cell line source(s)	NA
Authentication	NA
Mycoplasma contamination	NA
Commonly misidentified lines (See ICLAC register)	NA

Palaeontology and Archaeology

Specimen provenance	NA
Specimen deposition	NA

Dating methods | *NA*

☐ Tick this box to confirm that the raw and calibrated dates are available in the paper or in Supplementary Information.

Ethics oversight | *NA*

Note that full information on the approval of the study protocol must also be provided in the manuscript.

Animals and other research organisms

Policy information about studies involving animals; ARRIVE guidelines recommended for reporting animal research, and Sex and Gender in Research

Laboratory animals	All experiments were performed on mice with C57BL/6 background aged between 6-12 weeks.
Wild animals	*No wild animals were used in the study.*
Reporting on sex	*Mice of both sexes were included. It has been detailed in the "Method" section.*
Field-collected samples	*Field-collected samples were not used in the study.*
Ethics oversight	*All animal experiments were performed according to a UK Home Office licence (Project License 70/8646) and were reviewed by the animal welfare and ethical board of the University of Glasgow.*

Note that full information on the approval of the study protocol must also be provided in the manuscript.

Clinical data

Policy information about clinical studies

All manuscripts should comply with the ICMJE guidelines for publication of clinical research and a completed CONSORT checklist must be included with all submissions.

Clinical trial registration	*We used a subset of retrospective and anonymous molecular data from the FOCUS and PETACC3 clinical trials. These data were not prospectively used within the trial.*<br><br>*The FOCUS trial study was previously registered as an International Standard Randomised Controlled Trial, number ISRCTN 79877428. The PETACC3 trial study was previously registered under ClinicalTrials.gov Identifier NCT00026273.*
Study protocol	*FOCUS: https://www.isrctn.com/ISRCTN79877428*<br>*PETACC3: https://clinicaltrials.gov/ct2/show/NCT00026273*
Data collection	*NA*
Outcomes	*NA*

Dual use research of concern

Policy information about dual use research of concern

Hazards

Could the accidental, deliberate or reckless misuse of agents or technologies generated in the work, or the application of information presented in the manuscript, pose a threat to:

No	Yes	
☒	☐	Public health
☒	☐	National security
☒	☐	Crops and/or livestock
☒	☐	Ecosystems
☒	☐	Any other significant area

Experiments of concern

Does the work involve any of these experiments of concern:

No	Yes	
☒	☐	Demonstrate how to render a vaccine ineffective
☒	☐	Confer resistance to therapeutically useful antibiotics or antiviral agents
☒	☐	Enhance the virulence of a pathogen or render a nonpathogen virulent
☒	☐	Increase transmissibility of a pathogen
☒	☐	Alter the host range of a pathogen
☒	☐	Enable evasion of diagnostic/detection modalities
☒	☐	Enable the weaponization of a biological agent or toxin
☒	☐	Any other potentially harmful combination of experiments and agents

Plants

Seed stocks: *NA*

Novel plant genotypes: *NA*

Authentication: *NA*

ChIP-seq

Data deposition

☐ Confirm that both raw and final processed data have been deposited in a public database such as GEO.

☐ Confirm that you have deposited or provided access to graph files (e.g. BED files) for the called peaks.

Data access links
May remain private before publication.: *NA*

Files in database submission: *NA*

Genome browser session
(e.g. UCSC): *NA*

Methodology

Replicates: *NA*

Sequencing depth: *NA*

Antibodies: *NA*

Peak calling parameters: *NA*

Data quality: *NA*

Software: *NA*

Flow Cytometry

Plots

Confirm that:

☐ The axis labels state the marker and fluorochrome used (e.g. CD4-FITC).

☐ The axis scales are clearly visible. Include numbers along axes only for bottom left plot of group (a 'group' is an analysis of identical markers).

☐ All plots are contour plots with outliers or pseudocolor plots.

☐ A numerical value for number of cells or percentage (with statistics) is provided.

Methodology

Sample preparation	*NA*
Instrument	*NA*
Software	*NA*
Cell population abundance	*NA*
Gating strategy	*NA*

☐ Tick this box to confirm that a figure exemplifying the gating strategy is provided in the Supplementary Information.

Magnetic resonance imaging

Experimental design

Design type	*NA*
Design specifications	*NA*
Behavioral performance measures	*NA*

Acquisition

Imaging type(s)	*NA*
Field strength	*NA*
Sequence & imaging parameters	*NA*
Area of acquisition	*NA*

Diffusion MRI ☐ Used ☐ Not used

Preprocessing

Preprocessing software	*NA*
Normalization	*NA*
Normalization template	*NA*
Noise and artifact removal	*NA*
Volume censoring	*NA*

Statistical modeling & inference

Model type and settings	*NA*
Effect(s) tested	*NA*

Specify type of analysis: ☐ Whole brain ☐ ROI-based ☐ Both

Statistic type for inference (See Eklund et al. 2016)	*NA*
Correction	*NA*

Models & analysis

n/a	Involved in the study
☒	☐ Functional and/or effective connectivity
☒	☐ Graph analysis
☒	☐ Multivariate modeling or predictive analysis

Functional and/or effective connectivity: *NA*

Graph analysis: *NA*

Multivariate modeling and predictive analysis: *NA*
